# Winding Tensor Approach for the Analytical Computation of the Inductance Matrix in Eccentric Induction Machines

**DOI:** 10.3390/s20113058

**Published:** 2020-05-28

**Authors:** Javier Martinez-Roman, Ruben Puche-Panadero, Angel Sapena-Bano, Manuel Pineda-Sanchez, Juan Perez-Cruz, Martin Riera-Guasp

**Affiliations:** Institute for Energy Engineering, Universitat Politècnica de València, Camino de Vera s/n, 46022 Valencia, Spain; jmroman@die.upv.es (J.M.-R.); rupucpa@die.upv.es (R.P.-P.); asapena@die.upv.es (A.S.-B.); juperez@die.upv.es (J.P.-C.); mriera@die.upv.es (M.R.-G.)

**Keywords:** induction machines, inductance tensor, winding tensor approach, fault diagnosis, mixed eccentricity, winding asymmetries, discrete Fourier transform

## Abstract

Induction machines (IMs) are critical components of many industrial processes, what justifies the use of condition-based maintenance (CBM) systems for detecting their faults at an early stage, in order to avoid costly breakdowns of production lines. The development of CBM systems for IMs relies on the use of fast models that can accurately simulate the machine in faulty conditions. In particular, IM models must be able to reproduce the characteristic harmonics that the IM faults impress in the spatial waves of the air gap magneto-motive force (MMF), due to the complex interactions between spatial and time harmonics. A common type of fault is the eccentricity of the rotor core, which provokes an unbalanced magnetic pull, and can lead to destructive rotor-stator rub. Models developed using the finite element method (FEM) can achieve the required accuracy, but their high computational costs hinder their use in online CBM systems. Analytical models are much faster, but they need an inductance matrix that takes into account the asymmetries generated by the eccentricity fault. Building the inductance matrix for eccentric IMs using traditional techniques, such as the winding function approach (WFA), is a highly complex task, because these functions depend on the combined effect of the winding layout and of the air gap asymmetry. In this paper, a novel method for the fast and simple computation of the inductance matrix for eccentric IMs is presented, which decouples the influence of the air gap asymmetry and of the winding configuration using two independent tensors. It is based on the construction of a primitive inductance tensor, which formulates the eccentricity fault using single conductors as the simplest reference frame; and a winding tensor that converts it into the inductance matrix of a particular machine, taking into account the configuration of the windings. The proposed approach applies routine procedures from tensor algebra for performing such transformation in a simple way. It is theoretically explained and experimentally validated with a commercial induction motor with a mixed eccentricity fault.

## 1. Introduction

Induction machine (IM) maintenance, integrated in condition-based maintenance (CBM) systems [1,2,3,4,5], is a field of growing industrial interest, due to its widespread use in production lines, electrical vehicles, wind generators, etc. The failure of an IM can cause huge losses, due to unexpected breakdowns of machines and supply systems. To be responsive, CBMs must be able to operate on-line, in a non-invasive way, so that any fault can be detected in an incipient state and corrective measures can be deployed before the fault gets worse [6,7,8,9,10,11]. This requires fast and simple fault diagnostic techniques [12], that can be implemented in embedded field devices, such as digital signal-processors (DSPs) or field-programmable arrays (FPGAs). One of such diagnostic techniques relies on the design of sliding mode observers (SMO) for observing IM states obtained from the healthy and faulty model. In [13], an SMO that uses only input quantity information for on-line broken rotor bar detection is proposed, and in [14], a high-order SMO is designed for detecting inter-turn short circuit faults in IMs. Recent developments [15,16] in this field propose the use of a reduced-order SMO for fault estimation, which is able to simultaneously obtain the exact estimation of state, actuator faults, sensor faults, and extra disturbances. In [16], a novel approach is proposed without employing the equivalent output error injection technology, to overcome the problem of the traditional SMO in application to Markovian jump systems, and in [15], a new proposal is made for avoiding the sliding surface switching problem. Another diagnostic technique is to run an electromechanical model of the machine [17,18,19,20] and compare the simulation outputs (currents and voltages) with the quantities measured at the machine terminals. Divergences between the predicted and the measured values are an indicator of a possible fault, especially if these differences increase over time.

The IM models needed in the aforementioned diagnostic techniques can be built using the finite elements method (FEM) with a very high accuracy [21,22,23], but FEM demands huge computing resources, in terms of time and memory, which hinders its use in low-power embedded units. A faster and leaner alternative is to use analytical models [23,24] that can reproduce the characteristic harmonics induced in the current by a given fault. Another diagnostic area in which IM models are used is in the training of neural networks or expert systems for fault diagnosis [25,26,27,28,29,30,31], which need thousands of tests performed under different working conditions with controlled degrees of a machine fault. In this area, again, the speed of analytical models can give them a decisive advantage over FEM models.

Different IM analytical models for fault diagnosis have been proposed in the technical literature, based on the machine equations expressed in different coordinate systems, such as d−q, revolving fields, etc. These models rely on the calculation of the self and mutual inductances between all the machines phases, and their derivatives, as a function of the rotor position. This is a complex, non-linear function, which depends on the windings configurations, and on the rotor position. Besides, in case of a faulty machine, the air gap length or the configuration of the windings may become asymmetrical, making it difficult to use labour-saving procedures that are valid only for symmetrical conditions. In particular, the eccentricity fault [32,33] gives rise to a non-uniform air gap length, which becomes a function of the angular coordinate. Moreover, this function can be different for each rotor position [34]. To overcome this difficulty, the analytical methods for calculating the inductance matrix commonly apply the simplification of considering a sinusoidal distribution of the spatial waves in the air-gap, thus limiting the calculation of the inductances to its fundamental harmonic component. Nevertheless, complex interactions between spatial and time harmonics are present in a faulty machine, but are missing in models restricted to the fundamental component.

Several approaches have been used in the technical literature for obtaining the inductance matrix needed in analytical models. Its components can be determined by direct measurements, as in [35,36], or computed numerically. FEM models have been used for inductance computation in [23], and in [37] a FEM model is combined with a Preisach model for iron loss evaluation. An alternative is to use analytical methods for inductance computation. In [38] a review of the existing methods for the analytical computation of self and mutual inductances in a rotating electrical machine are described, and a new approach based on energy method is presented. A drawback of the analytical methods is that they do not take into account the saturation, the iron path, or the end leakage inductances. In [39] these factors have been simulated via modified air gap length functions.

Instead of a direct, analytical computation of the inductances of phases with a complex winding layout, a successful approach is to start with the inductances of elementary coils, and to combine them via connection matrices to obtain the phase inductances [40]. This approach has been followed also in the winding function approach (WFA) [39]. In [41] the WFA has been combined with a conformal transformation in order to take into account air gap length variations due to the slots. A drawback of these methods is that they need complex winding functions, which depend on the relative position of the coils and the rotor position. Moreover, these functions depend on the combined effect of the winding layout and of the air gap asymmetry, which makes their computation a highly complex task.

In this work, this line of research is followed, with two fundamental novelties: replacing the coils by the conductor as the basic, most simple winding unit, and using routine tensor algebra for the analytical computation of the inductance matrix. This approach allows decoupling the combined effects of the air gap asymmetry and of the winding configuration in the calculation of the inductance matrix, greatly simplifying its analytical computation. The proposed method for calculating the inductance matrix is developed in two steps:

1. First, a primitive inductance tensor is calculated in a reference frame that consists of a thin cylindrical sheet of a high number of parallel bars, statically fixed to the air gap. This can be considered, as [42] states, as a canonical coordinate system, in which the components of the primitive inductance tensor are the same for every IM, except for a scaling factor.
2. The primitive inductance tensor is transformed into the final one via a winding tensor [40], which contains the current-sheet generated by each phase when fed by a unit current, using routine tensor algebra procedures.

The proposed approach neatly decouples the geometrical configuration of the machine air gap (which can be asymmetrical), represented in the primitive inductance tensor, and the configuration of the phase windings (which can be arbitrarily complex), represented in the winding tensor. Both tensors are defined analytically in an independent way, which simplifies their formulation. Their combined effect, obtained using routine tensor algebra operations, gives the final IM inductance matrix in a simple and fast way, which may be denoted as the winding tensor approach (WTA).

The structure of the paper is the following one. In Section 2, the analytical model of the IM used in this work, in a natural coordinate system, is presented. The analytical computation of the inductance matrix that appears in this model is developed in Section 3 for the case of a healthy and an eccentric IM, using tensor algebra. In Section 4 tensor algebra is applied again to take into account different phase connections, as those imposed by a squirrel cage rotor. An experimental validation of the proposed approach is carried out using a commercial IM motor with a provoked mixed eccentricity fault. This motor is first simulated in Section 5, and the results are compared with those obtained from the experimental tests in Section 6. Finally, Section 7 presents the conclusions of this work.

## 2. Analytical Model of the IM Using a Natural Coordinate System

Let’s consider a generic IM with $n_{s}$ stator phases and $n_{r}$ rotor phases, with a total number of phases $n=n_{s}+n_{r}$. Among the multiple coordinate systems that can be used for obtaining an analytical model of the IM (dq, symmetrical components, revolving fields, etc.), a natural coordinate system has been chosen in this work: each stator phase ($s_{1},s_{2}\ldots s_{n_{s}}$) has its own axis as a stationary coordinate axis, and each rotor phase ($r_{1},r_{2}\ldots r_{n_{r}}$) has its own axis as a moving coordinate axis, attached to the phase conductors. All the *n* phase currents in this coordinate system are considered to be independent variables, thus defining an *n*-dimensional space. From the point of view of the IM simulation, this choice has the advantage of directly giving the phase currents, without needing any further transformation.

In the natural coordinate system, two equations are needed to model the IM operation [40,43]

(1) $$\begin{array}{cccc} • & \;\mathrm{Equation}\;\mathrm{of}\;\mathrm{voltage}: & \; & e=Ri+L\frac{di}{dt}+i\frac{dL}{d\theta }\dot{\theta }\\ • & \;\mathrm{Equation}\;\mathrm{of}\;\mathrm{torque}: & \; & T=R_{\theta }\dot{\theta }+J\frac{d\dot{\theta }}{dt}-\frac{1}{2}i^{t}\frac{dL}{d\theta }i \end{array}$$

where the subscript ‘${}^{t}$’ stands for the transpose operator. The quantities that appear in (1) are the following ones:

- i is the current tensor. Its components are the instantaneous current in each winding $i={\left[i_{1},i_{2},\ldots ,i_{n}\right]}^{t}$.
- e is the voltage tensor. Its components are the instantaneous terminal voltages applied to each winding $e={\left[e_{1},e_{2},\ldots ,e_{n}\right]}^{t}$.
- R is the resistance tensor. Its components are the resistances of all windings. It is a symmetrical dyadic tensor, an square array of $n^{2}$ constant components.
- L is the inductance tensor. Its components are the self and mutual inductances of all windings along the electrical axes. It is a symmetrical dyadic tensor, an square array of $n^{2}$ elements. It can be expressed as the sum of two components, one with the inductances corresponding to the main flux linkages $L_{m}$, and other with the leakage inductances $L_{\sigma }$, as
  (2) $$L=L_{m}+L_{\sigma }$$
  End turns, end rings, and slot leakage inductances, included in the $L_{\sigma }$ matrix, need to be pre-calculated, as usual in the technical literature, where explicit expressions for these inductances can be found in [44,45,46]. This work deals only with the analytical computation of $L_{m}$ in (2). Linear behavior of the iron material will be assumed, as in [47]. This limitation of the analytical model can be overcome using a modified air gap length function to take into the saturation, as in [39].
- The rest of the terms that appear in (1) are the instantaneously applied shaft torque *T*, the frictional resistance of the shaft $R_{\theta }$, and the moment of inertia *J*.

The equation of voltage in (1) can be expressed in a more condensed form making use of the tensor of flux linkages of the IM phases, φ=Li. Besides, neglecting the frictional resistance of the shaft ($R_{\theta }$ = 0) in the equation of torque in (1), the set of electro-mechanical equations of the IM, in the natural coordinate system, is given by

(3) $$\left\{\begin{array}{ccc} e & = & Ri+\frac{d\varphi }{dt}\\ T & = & J\frac{d\dot{\theta }}{dt}-\frac{1}{2}i^{t}\frac{dL}{d\theta }i \end{array}\right.$$

**Figure 1:**
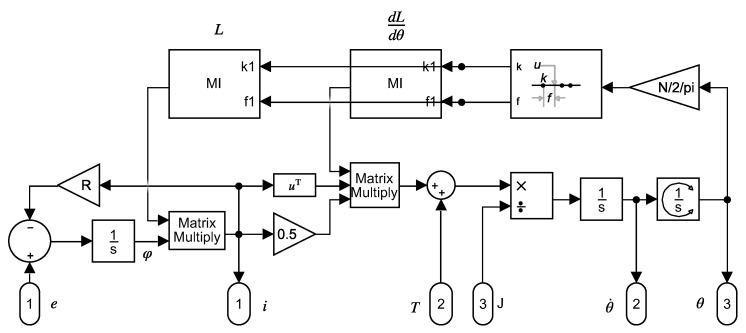
Analytical model that implements (3) in Simulink.

An implementation of (3) in a Simulink model is shown in Figure 1. The mutual inductances between the stator and rotor phases in $L_{m}$ (2) depend on the rotor position, and must be updated at each step of the simulation.

### Transformation of the Coordinate System

The quantities i, e, R and L are tensors, that is, if a different coordinate system is chosen (for example, the hypothetical axis of symmetrical components, or a stationary dq coordinate system), these quantities remain invariant. Only their components (currents, voltages, self and mutual inductances, etc.) in the new coordinate system will be transformed, in the same way that an invariant vector can have different components under different coordinate systems, in spite of not changing neither its modulus nor its orientation. In particular, if the current tensor is expressed in a coordinate system different than the natural one, its new components $i^{\prime }$ would be different than the old ones, i. Nevertheless, if the matrix C of the coordinate transformation is given, then the relation between the old components and the new ones can be expressed as

(4) $$i=Ci^{\prime }$$

and the transformation law of the rest of tensors e, R and L is given, applying tensor algebra, by

(5) $$\begin{array}{ccc} e^{\prime } & = & C^{t}e\\ L^{\prime } & = & C^{t}LC\\ R^{\prime } & = & C^{t}RC \end{array}$$

where $C^{t}$ stands for the transpose of matrix C.

## 3. Computation of the Mutual Inductance Matrix Using Tensor Algebra

Neglecting the iron saturation and losses, mutual inductances depend only on the geometry of the system [48]. Therefore, their computation is done in this work in the spatial domain of the air gap, using the current-sheet generated by the phase currents i. The steps of this approach are:

1. Definition of the canonical coordinate system for representing the current-sheet distribution along the air gap periphery. In this system, the components of the tensors i and $L_{m}$ are independent of the connections of the phase conductors.
2. Calculation of the current-sheet from the phase currents i. The winding tensor contains the connections between the conductors of the phases, which can be arbitrarily complex.
3. Definition of the primitive inductance tensor in the canonical coordinate system, which is independent of the layout of the winding, and the same for every IM, apart from a scaling factor.
4. Transformation of the inductance tensor to the natural coordinate system using tensor algebra (5).

### 3.1. The Components of the Current Tensor

The components of the current tensor are obtained first in the canonical coordinate system, where they are independent of the connections of the phase conductors, and after transformed to the natural coordinate system, using a winding tensor that represents the windings layout.

#### 3.1.1. The Current Tensor in the Canonical Coordinate System

**Figure 2:**
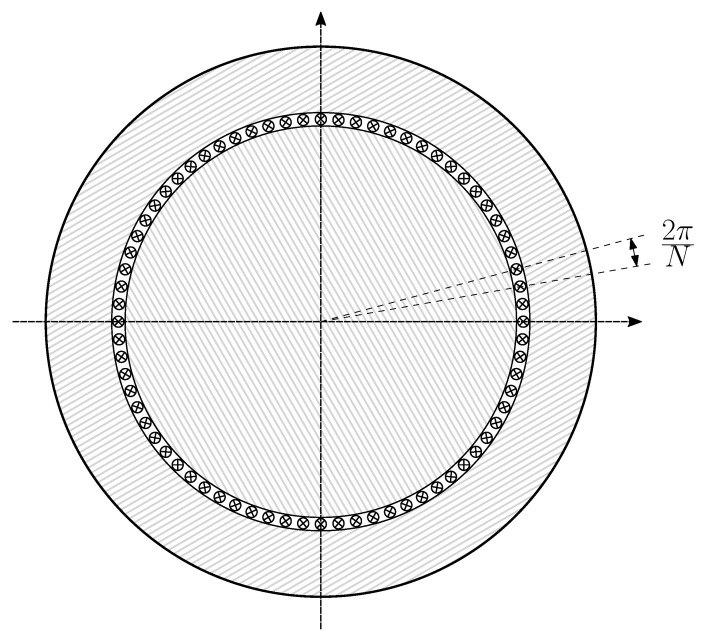
Coordinate system constituted by *N* independent conductors. A current-sheet with up to N/2 spatial harmonics can be represented in this system. The *N* components of the current tensor in this system, $i_{c}$, are the currents through each conductor.

The physical representation of the current tensor i in rotating electrical machines is a current-sheet distributed along the air gap periphery [43]. The most suitable coordinate system to represent it consists in a thin cylindrical sheet of *N* parallel bars [40], statically placed at the air gap, as shown in Figure 2. The value of *N* must be high to achieve a high spatial resolution (*N* = 3600 in this work). The width of each individual bar is assumed to be 2π/N, while its height is considered negligible. An electrical coordinate axis is attached to each conductor, thus defining a *N* dimensional space in which any current-sheet can be represented with up to N/2 spatial harmonics. This *N* dimensional space is spanned by a basis with *N* elements, given by

(6) $$\left\{\begin{array}{ccc} u_{c1} & = & {\left[1,0,0,\ldots ,0\right]}^{t}\\ u_{c2} & = & {\left[0,1,0,\ldots ,0\right]}^{t}\\ & \ldots & \\ u_{\mathrm{cN}} & = & {\left[0,0,0,\ldots ,1\right]}^{t} \end{array}\right.$$

where the *k*th basis element $u_{\mathrm{ck}}$ has all components equal to 0 except the *k*th that is 1. The vectors $u_{\mathrm{ck}}$ are unitary and orthogonal, and they form an ordered basis, which is called the standard or canonical basis. In this basis, the current-sheet can be represented as a linear combination of the basis elements as

(7) $$i_{c}=\sum_{k=1}^{N}i_{ck}\cdot u_{\mathrm{ck}}$$

where $i_{ck}$ the current in conductor *k*. That is, the components of the current tensor in this coordinate system, $i_{c}$, are simply the *N* currents through the *N* independent conductors

(8) $$i_{c}={\left[i_{c_{1}},i_{c_{2}},\ldots ,i_{c_{N}}\right]}^{t}$$

#### 3.1.2. Transformation of the Current Tensor to Natural Coordinates

The current-sheet $i_{c}$ (8) can be also expressed in the natural coordinate system, using the *n* phase currents as independent variables. This *n* dimensional space is spanned by a basis formed by *n* basis vectors, $n_{s}$ stator and $n_{r}$ rotor vectors. In this basis, the current-sheet $i_{c}$ can be represented as a linear combination of the new basis vectors as

(9) $$i_{c}=\sum_{k=1}^{n}i_{k}\cdot z_{k}$$

where $i_{k}$ represents the current in phase *k*. Each basis vector in (9) has *N* components, which for the *k*th basis vector $z_{k}$ are the ampere-turns generated by phase *k* at each angular interval of Figure 2, when fed with a unit dc current. This value coincides with the number of conductors of phase *k* in each interval, with a ± sign corresponding to the direction of the current.

(10) $$\left\{\begin{array}{ccc} z_{1} & = & {\left[z_{11},z_{21},z_{31},\ldots ,z_{N1}\right]}^{t}\\ z_{2} & = & {\left[z_{12},z_{22},z_{32},\ldots ,z_{N2}\right]}^{t}\\ & \vdots & \\ z_{n} & = & {\left[z_{1n},z_{2n},z_{3n},\ldots ,z_{Nn}\right]}^{t} \end{array}\right.$$

The number of basis vectors in this new coordinate system (10) is much lower than in the primitive coordinate system (6). Nevertheless, they are neither unitary nor orthogonal.

The new vector basis (10) can be expressed in the canonical basis (6) as

(11) $$z_{k}=\sum_{l=1}^{N}z_{kl}\cdot u_{l}$$

Using (10) and (11), (9) becomes

(12) $$i_{c}=\sum_{k=1}^{n}\sum_{l=1}^{N}i_{k}\cdot z_{kl}\cdot u_{l}$$

This coordinate transformation can be formulated using a (N×n) transformation matrix $C_{c}$ as (4)

(13) $$i_{c}=C_{c}i$$

where the columns of $C_{c}$ are the new basis vectors (10),

(14) $$C_{c}=\left[\begin{array}{cccc} z_{11} & z_{12} & \cdots & z_{1n}\\ z_{21} & z_{22} & \cdots & z_{2n}\\ \vdots & \vdots & \ddots & \vdots \\ z_{N1} & z_{N2} & \cdots & z_{Nn} \end{array}\right]$$

The transformation matrix $C_{c}$ represents the current constraints imposed by the connections between the conductors of each winding. Therefore, as Kron states in [40], this particular transformation matrix can be considered as one aspect of the transformation tensor $C_{c}$, that will be referred to as the winding tensor. Its (i,j) element contains the number of phase conductors of phase *j* in an angular interval π/2N, centered at $i\cdot \frac{2\pi }{N}$. This winding tensor must be obtained for the *N* possible angular positions of the rotor ($\theta_{k}=k\cdot \frac{2\pi }{N}$, with k=0,…,N−1). Nevertheless, the columns of $C_{c}$ corresponding to the rotor phases for a given rotor position $\theta_{k}$ are the same as the columns defined with the rotor at the origin ($\theta_{0}=0$), but rotated *k* positions.

#### 3.1.3. The Winding Tensor for Phases with the Same Configuration

In (14) no restrictions are imposed on the connections of the conductors of each phase, which can be arbitrarily complex, as in the case of asymmetrical windings (turn-to-turn short circuits, broken bars, etc.). Nevertheless, in case of a healthy machine, the configuration of all the phases of a particular winding (stator or rotor) is the same. Therefore, the vector column of $C_{c}$ corresponding to the *k*th stator phase is equal to the vector column of the first stator phase, but rotated $k\cdot N/n_{s}$ elements to the bottom. The same applies to the rotor phases, but in this case the rotation is $k\cdot N/n_{r}$ positions.

### 3.2. The Components of the Mutual Inductance Tensor

The components of the mutual inductance tensor will be obtained first in the canonical coordinate system, where they are independent of the connections of the phase conductors, and then transformed to the natural coordinate system, using the winding tensor.

#### 3.2.1. The Primitive Inductance Tensor

In the canonical coordinate system (6), the mutual inductance tensor, $L_{\mathrm{mc}}$, is a N×N square matrix

(15) $$L_{\mathrm{mc}}=\left[\begin{array}{cccc} L_{mc_{11}} & L_{mc_{12}} & \cdots & L_{mc_{1N}}\\ L_{mc_{21}} & L_{mc_{22}} & \cdots & L_{mc_{2N}}\\ \vdots & \vdots & \ddots & \vdots \\ L_{mc_{N1}} & L_{mc_{N2}} & \cdots & L_{mc_{NN}} \end{array}\right]$$

whose component (i,j), $L_{mc_{ij}}$, is the mutual partial inductance [24] between the conductors placed at positions $i\cdot \frac{2\pi }{N}$ and $j\cdot \frac{2\pi }{N}$. The tensor $L_{\mathrm{mc}}$ (15) will be denoted as the primitive inductance tensor.

#### 3.2.2. The Primitive Inductance Tensor of a Non-Eccentric IM

In case of IMs with uniform air gap, as represented in Figure 2, $L_{mc_{ij}}$ depends only on the angular separation between conductors *i* and *j*, as given in [49]

(16) $$L_{\mathrm{mc}}\left(i,j\right)=L_{mc_{ij}}=\frac{\mu_{0}\cdot l\cdot r\cdot \pi }{g}\cdot {\left(\frac{1}{2}-\frac{\left|i-j\right|}{N}\right)}^{2}$$

where $\mu_{0}=4\pi \times 10^{-7}$ T·m·A^−1^, *l* is the effective length of the stator bore, *r* is the radius at the center of the air gap, and *g* is the air gap length.

From (16), the components of $L_{\mathrm{mc}}$ are the same for every IM, except for the scaling factor $\frac{\mu_{0}\cdot l\cdot r\cdot \pi }{g}$, which depends only on the geometrical dimensions of the machine, *l*, *r* and *g*. Besides, $L_{\mathrm{mc}}$ is a circulant, symmetrical matrix, where every column vector is obtained by rotating one element to the bottom of the preceding column vector.

#### 3.2.3. The Primitive Inductance Tensor of an Eccentric IM

**Figure 3:**
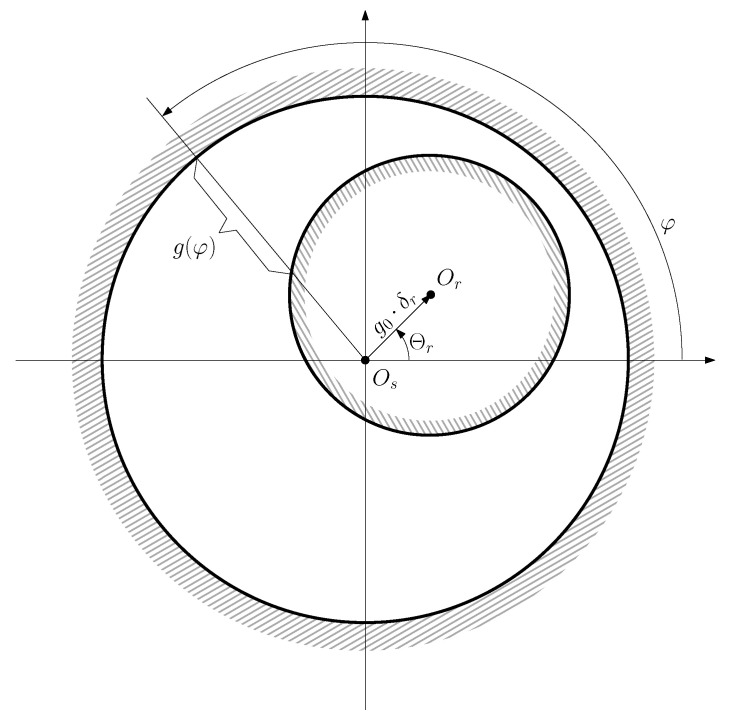
Air gap length g(φ) of an eccentric machine as a function of the angular coordinate φ, measured in the canonical coordinate system. The function g(φ) is fully defined by the position of the rotor center $O_{r}$ with respect to the stator center $O_{s}$.

In the case of rotor eccentricity the air gap length is not uniform, because the rotor center $O_{r}$ does not coincide with the stator center $O_{s}$, as shown in Figure 3.

**Figure 4:**
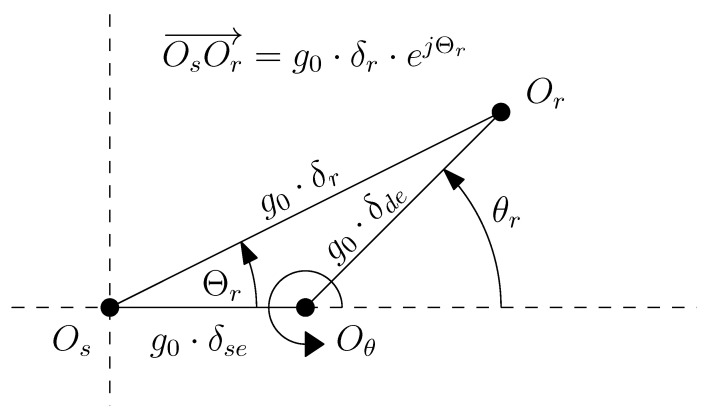
Position of the rotor center ($O_{r}$), the stator center ($O_{s}$), and the axis of rotation ($O_{\theta }$) in a coordinate system fixed to the stator, in case of an IM with static ($\delta_{se}$) and dynamic ($\delta_{de}$) eccentricity.

From Figure 3, the position of the rotor center can be represented using its radial coordinate, $\delta_{r}\cdot g_{0}$, and its angular coordinate $\Theta_{r}$, as

(17) $$\vec{O_{s}O_{r}}=g_{0}\cdot \delta_{r}\cdot e^{j\Theta_{r}}\;0\leq \delta_{r}<1,\;0\leq \Theta_{r}<2\pi$$

where $g_{0}$ is the air gap length of the IM without any eccentricity, and $\delta_{r}$ is the degree of eccentricity ($0\leq \delta_{r}<1$). Additionally, the coordinates ($g_{0}\cdot \delta_{r}$, $\Theta_{r}$) of the rotor center depend on the angular position of the rotor $\theta_{r}$, and the degree of static $\delta_{se}$ and dynamic $\delta_{de}$ eccentricity of the machine (see Figure 4), as

(18) $$\Theta_{r}\left(\theta_{r}\right)=\tan^{-1}\left(\frac{\delta_{de}\sin\left(\theta_{r}\right)}{\delta_{se}+\delta_{de}\cos\left(\theta_{r}\right)}\right)$$

(19) $$\delta_{r}\left(\theta_{r}\right)=\sqrt{{\delta_{se}}^{2}+{\delta_{de}}^{2}+2\delta_{se}\delta_{de}\cos\left(\theta_{r}\right)}$$

where $\theta_{r}$ represents the angle of rotation of the machine rotor.

For computing the inductance matrix, the inverse of the air gap length function is needed to obtain the permeance function of the machine. It can be fully defined in terms of the coordinates of the rotor center (17) as [23]

(20) $$g{\left(\varphi ,\Theta_{r},\delta_{r}\right)}^{-1}=g_{0}^{-1}\cdot \left(A_{0}+\sum_{m=1}^{n_{t}}A_{m}\cdot \cos\left(m(\varphi -\Theta_{r})\right)\right)$$

where

(21) $$A_{0}=\frac{1}{\sqrt{1-{\delta_{r}}^{2}}}\;\;\;\;\;\;\;\;\;\;\;\;\;\;\;A_{m}=2{\left(\frac{1-\sqrt{1-{\delta_{r}}^{2}}}{\sqrt{1-{\delta_{r}}^{2}}}\right)}^{m}\;m=1\ldots n_{t}$$

It is worth mentioning that only the first term of the series in (20) has been used in [50,51,52,53], and two terms in [54]. In this paper, (20) can take into account a generic number $n_{t}$ of terms, where the value of $n_{t}$ can be chosen to achieve the desired precision.

Each component (*i*,*j*) of the induction matrix $L_{mc_{ij}}$ in an eccentric IM depends not only on the angular separation between conductors *i* and *j*, but also on their absolute position and on the position of the rotor center, whose coordinates ($g_{0}\cdot \delta_{r}$, $\Theta_{r}$) are, in turn, functions of the rotor angular position (18), (19). The analytical expression of $L_{mc_{ij}}$, for a given rotor position $\theta_{k}=k\cdot \frac{2\pi }{N}$, can be expressed as [24]

(22) $$L_{\mathrm{mc}}\left(i,j\right){|}_{k}=\frac{\mu_{0}lr}{g_{0}}\cdot \Lambda \left(i\frac{2\pi }{N},j\frac{2\pi }{N},\Theta_{r}\left(k\frac{2\pi }{N}\right),\delta_{r}\left(k\frac{2\pi }{N}\right)\right)$$

where

(23) $$\begin{array}{c} \Lambda \left(\alpha ,\varphi ,\Theta_{r},\delta_{r}\right)=\frac{A_{0}}{4\pi }{\left(\varphi -\alpha \right)}^{2}+\sum_{m=1}^{n_{t}}\frac{A_{m}}{2\pi }\left(\frac{\left(\varphi -\alpha \right)\sin\left(m(\varphi -\Theta_{r})\right)}{m}+\frac{\cos\left(m(\varphi -\Theta_{r})\right)}{m^{2}}\right)-\\ \left(\frac{1}{2}-K\left(\alpha ,\Theta_{r},\delta_{r}\right)\right)\cdot \left(A_{0}\left(\varphi -\alpha \right)+\sum_{m=1}^{n_{t}}A_{m}\frac{\sin\left(m(\varphi -\Theta_{r})\right)}{m}\right) \end{array}$$

and

(24) $$K\left(\alpha ,\Theta_{r},\delta_{r}\right)=\sum_{m=1}^{n_{t}}\frac{A_{m}}{2\pi A_{0}}\frac{\sin(m\left(\Theta_{r}-\alpha \right))}{m}$$

From (22), the components of $L_{\mathrm{mc}}$ are the same for every IM with a given degree of static and dynamic eccentricity, except for the scaling factor $\frac{\mu_{0}\cdot l\cdot r\cdot \pi }{g_{0}}$, which depends only on the geometrical dimensions of the machine.

It is worth remarking that the primitive inductance tensor $L_{\mathrm{mc}}$ includes the effect of the air gap asymmetry generated by the mixed eccentricity fault, but is independent of the winding configuration, because it has been obtained using the conductor as the basic unit. Therefore, it is valid for any IM, except for a scaling factor. This leads to a great simplification compared with other existing methods, such as the WFA, which rely on winding functions whose definition depends both on the air gap asymmetry and on the configuration of the winding coils.

#### 3.2.4. Transformation of the Inductance Tensor to Natural Coordinates

In the natural coordinate system, for each rotor position, the inductance tensor $L_{m}$ in (2) can be obtained from the primitive inductance tensor $L_{\mathrm{mc}}$, either considering healthy (16) or eccentric machine (22), and from the winding tensor $C_{c}$, as (5)

(25) $$L_{m}={C_{c}}^{t}L_{\mathrm{mc}}C_{c}$$

Again, at this point it is worth remarking that the transformation from the primitive inductance tensor $L_{\mathrm{mc}}$, which includes only the effect of the air gap asymmetry generated by the mixed eccentricity fault, into the final inductance matrix $L_{m}$, taking into account the configuration of the windings, is a routine tensor algebra operation (25) that simply consists in multiplying the primitive inductance tensor by the winding tensor $C_{c}$ (14) (and its transpose). The winding tensor has been defined without any relation to the air gap asymmetry, simply indicating the number and direction of the winding conductors at each interval of the rotor and stator periphery.

This advantage can be further exploited to introduce the effect of winding related faults in the model through the winding tensor $C_{c}$, such as inter-turn short circuits or phase asymmetries. This would allow the analysis of combined eccentricity and winding faults with a small increase in analytical and computational complexity. Due to space constraints, this approach has not been considered in this work, and will be presented in a future one.

## 4. Additional Current Constraints Imposed by Phase Connections

In (3) the phase currents i are independent variables. Nevertheless, the connections between the phases can make some currents dependent on others. These constraints can be expressed as a connection tensor $C_{i}$, which relates the original, independent phase currents i (before considering the interconnections between the phases), with the new ones $i^{\prime }$, including the constraints introduced by the phase connections as

(26) $$i=C_{i}i^{\prime }$$

As the transformation given by $C_{i}$ is holonomic, because its components do not depend on the rotor position, (1) remains valid when expressed in terms of the new, reduced quantities $i^{\prime }$, $e^{\prime }$, $L^{\prime }$ and $R^{\prime }$, giving

(27) $$\begin{array}{c} \left\{\begin{array}{ccc} e^{\prime } & = & R^{\prime }i^{\prime }+L^{\prime }\frac{di^{\prime }}{dt}+i^{\prime }\frac{dL^{\prime }}{d\theta }\dot{\theta }\\ T & = & R_{\theta }\dot{\theta }+J\frac{d\dot{\theta }}{dt}-\frac{1}{2}i^{\prime t}\frac{dL^{\prime }}{d\theta }i^{\prime } \end{array}\right. \end{array}$$

where $e^{\prime }={C_{i}}^{t}e$, $R^{\prime }={C_{i}}^{t}RC_{i}$, and $L^{\prime }={C_{i}}^{t}LC_{i}$.

A particular example of current constraints in squirrel cage IMs are those imposed by the physical configuration of the rotor cage, which are analyzed in the following Section.

### Current Constraints in a Squirrel Cage IM

In the analytical model of a squirrel cage IM, the $n_{s}$ stator phases are considered as independent electrical circuits in (3), without any current constraints. Therefore, the resistance and leakage tensors of the stator winding are square matrices of size $n_{s}\times n_{s}$, whose diagonal terms are equal to the stator phase resistances, $R_{s}$, and to the phase leakage inductances, $L_{\sigma s}$, respectively. All the terms outside the diagonals are zero. If all the stator phases have the same configuration then

(28) $$R_{s}=\left[\begin{array}{ccc} R_{s} & & 0\\ & \ddots & \\ 0 & & R_{s} \end{array}\right]$$

(29) $$L_{\sigma s}=\left[\begin{array}{ccc} L_{\sigma s} & & 0\\ & \ddots & \\ 0 & & L_{\sigma s} \end{array}\right]$$

where $R_{s}$ and $L_{\sigma s}$ are the resistance and the leakage inductance of a stator phase, respectively.

**Figure 5:**
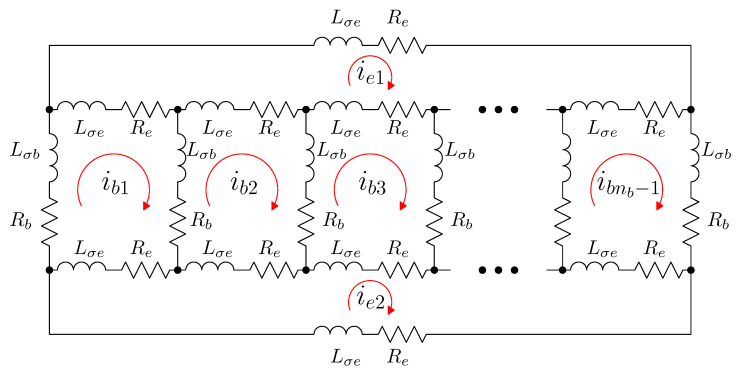
Rotor loops in a squirrel cage rotor of $n_{b}$ bars. There are $n_{b}-1$ rotor loops, formed by two consecutive bars, whose currents ($i_{b1}$ to $i_{bn_{b}-1}$) are coupled to each other and to the stator currents through their mutual inductances. Besides, there are two end ring loops, whose currents ($i_{e1}$ and $i_{e2}$) do not couple with the stator currents, and couple with the other rotor loop currents only through the end ring leakage inductances and the end ring resistances ($L_{\sigma e}$ and $R_{e}$ respectively).

The electric circuit of the squirrel cage rotor, with $n_{b}$ bars, can be built using $n_{b}$ rotor loops (each loop formed by two consecutive rotor bars), plus two additional loops corresponding to the end rings [55,56], as represented in Figure 5. The currents in the loops formed by two consecutive bars ($i_{b1}$ to $i_{bn_{b}}$) are coupled to each other and to the stator currents through their mutual inductances. On the contrary, the end ring loop currents ($i_{e1}$ and $i_{e2}$ in Figure 5) do not couple with the stator currents, and couple with the other rotor loop currents only through the end ring leakage inductances and the end ring resistances ($L_{\sigma e}$ and $R_{e}$ in Figure 5, respectively). Therefore, the resistance and leakage tensors of the squirrel cage rotor (with $n_{b}$ bars) are square matrices of size $\left(n_{b}+2\right)\times \left(n_{b}+2\right)$, due to the presence of the two extra loops formed by the end rings.

The resistance matrix of the rotor cage of Figure 5 is given by

(30) $$R_{b}=[\begin{array}{ccccccccc} R_{be} & -R_{b} & & & & & & -R_{e} & -R_{e}\\ -R_{b} & R_{be} & -R_{b} & & & & & -R_{e} & -R_{e}\\ & -R_{b} & R_{be} & -R_{b} & & 0 & & -R_{e} & -R_{e}\\ & & \ddots & \ddots & \ddots & & & \vdots & \vdots \\ & 0 & & -R_{b} & R_{be} & -R_{b} & & -R_{e} & -R_{e}\\ & & & & -R_{b} & R_{be} & -R_{b} & -R_{e} & -R_{e}\\ & & & & & -R_{b} & R_{be} & -R_{e} & -R_{e}\\ -R_{e} & -R_{e} & \ldots & -R_{e} & -R_{e} & -R_{e} & -R_{e} & n_{b}R_{e} & 0\\ -R_{e} & -R_{e} & \ldots & -R_{e} & -R_{e} & -R_{e} & -R_{e} & 0 & n_{b}\;R_{e} \end{array}]$$

with $R_{be}=2\left(R_{b}+R_{e}\right)$.

Nevertheless, it is advisable to reduce the set of rotor currents to those circulating in the loops that contain rotor bars, which are the only ones coupled through the mutual loop inductances $L_{m}$ (25). In this way, the tensor of rotor currents is reduced to

(31) $$i_{r}={\left[i_{b1},i_{b2},\ldots ,i_{bn_{b}-1}\right]}^{t}$$

This reduction can be achieved because there are two current constraints in the electrical circuit Figure 5. In effect, if the end ring loops mutual inductances with the rest of the rotor loops and with the stator phases are neglected, being mainly end winding flux linkages, the currents in the end ring loops can be expressed as:

(32) $$n_{b}\left(R_{e}i_{e1}+L_{\sigma e}\frac{d\;i_{e1}}{dt}\right)-\sum_{i=1}^{n_{b}-1}(R_{e}i_{bi}+L_{\sigma e}\frac{d\;i_{bi}}{dt})=0\;\Rightarrow \;i_{e1}=\frac{1}{n_{b}}\sum_{i=1}^{n_{b}-1}i_{bi}$$

(33) $$n_{b}\left(R_{e}i_{e2}+L_{\sigma e}\frac{d\;i_{e2}}{dt}\right)-\sum_{i=1}^{n_{b}-1}(R_{e}i_{bi}+L_{\sigma e}\frac{d\;i_{bi}}{dt})=0\;\Rightarrow \;i_{e2}=\frac{1}{n_{b}}\sum_{i=1}^{n_{b}-1}i_{bi}$$

These constraints can be formulated as in (26). The old set $i_{b}$ of $n_{b}+1$ rotor currents ($n_{b}-1$ current loops that include the bars plus two end ring currents) can be obtained from the new set $i_{r}$ of $n_{b}-1$ currents (just the current loops containing the bars), using (32) and (33), as

(34) $$\begin{array}{ccccc} \underset{︸}{\left[\begin{array}{c} i_{b1}\\ i_{b2}\\ \vdots \\ i_{bn_{b}-1}\\ i_{e_{1}}\\ i_{e_{2}} \end{array}\right]} & = & \underset{︸}{\left[\begin{array}{cccc} 1 & & & \\ & 1 & 0 & \\ & 0 & \ddots & \\ & & & 1\\ \frac{1}{n_{b}} & \frac{1}{n_{b}} & \ldots & \frac{1}{n_{b}}\\ \frac{1}{n_{b}} & \frac{1}{n_{b}} & \ldots & \frac{1}{n_{b}} \end{array}\right]} & \cdot & \underset{︸}{\left[\begin{array}{c} i_{b1}\\ i_{b2}\\ \vdots \\ i_{bn_{b}-1} \end{array}\right]}\\ i_{b} & = & C_{i} & \cdot & i_{r}\\ (n_{b}+1)\times 1 & & \left(n_{b}+1\right)\times \left(n_{b}-1\right) & & (n_{b}-1)\times 1 \end{array}$$

Using the circuit transformation tensor $C_{i}$ in (34), which transforms branch resistances into loop ones, as in [57,58], the resistance matrix of the rotor $R_{r}$ that includes the current constraints (32) and (33) is

(35) $$R_{r}={C_{i}}^{t}\cdot R_{b}\cdot C_{i}$$

giving the final result

(36) $$R_{r}=[\begin{array}{ccccccc} R_{be} & -R_{b} & & & & & \\ -R_{b} & R_{be} & -R_{b} & & & & \\ & -R_{b} & R_{be} & -R_{b} & & 0 & \\ & & \ddots & \ddots & \ddots & & \\ & 0 & & -R_{b} & R_{be} & -R_{b}\\ & & & & -R_{b} & R_{be} & -R_{b}\\ & & & & & -R_{b} & R_{be} \end{array}]-\frac{2R_{e}}{n_{b}}\cdot \left[\begin{array}{cccc} 1 & 1 & \ldots & 1\\ 1 & 1 & \ldots & 1\\ \vdots & \vdots & \ddots & \vdots \\ 1 & 1 & \ldots & 1 \end{array}\right]$$

The term $\frac{2R_{e}}{n_{b}}$ is small for a high value of $n_{b}$, which may justify considering only one end ring current loop, as in [59], or even to neglect both end ring current loops.

In a similar way, the matrix of rotor leakage inductances is given by

(37) $$\begin{array}{cc} L_{\sigma r} & =\left[\begin{array}{ccccccc} L_{\sigma be} & -L_{\sigma b} & & & & & \\ -L_{\sigma b} & L_{\sigma be} & -L_{\sigma b} & & & & \\ & -L_{\sigma b} & L_{\sigma be} & -L_{\sigma b} & & 0 & \\ & & \ddots & \ddots & \ddots & & \\ & 0 & & -L_{\sigma b} & L_{\sigma be} & -L_{\sigma b} & \\ & & & & -L_{\sigma b} & L_{\sigma be} & -L_{\sigma b}\\ & & & & & -L_{\sigma b} & L_{\sigma be} \end{array}\right]-\\ & -\frac{2L_{\sigma e}}{n_{b}}\cdot \left[\begin{array}{cccc} 1 & 1 & \ldots & 1\\ 1 & 1 & \ldots & 1\\ \vdots & \vdots & \ddots & \vdots \\ 1 & 1 & \ldots & 1 \end{array}\right] \end{array}$$

where $L_{\sigma be}=2\left(L_{\sigma b}+L_{\sigma e}\right)$.

Using (28), (29), (36) and (37), the final resistance and leakage inductance matrices that are used in the electromechanical equations of the healthy squirrel cage IM are

(38) $$R=\left[\begin{array}{cc} R_{s} & 0\\ 0 & R_{r} \end{array}\right]$$

(39) $$L_{\sigma }=\left[\begin{array}{cc} L_{\sigma s} & 0\\ 0 & L_{\sigma r} \end{array}\right]$$

## 5. Analytical Model of the Tested IM

In this Section, the analytical model of the commercial IM whose characteristics are given in Appendix A, which is used for the experimental validation of the proposed approach, is calculated considering two different motor conditions:

- Healthy conditions.
- Faulty conditions, with a mixed eccentricity fault, with 30% of static eccentricity ($\delta_{se}$ = 0.3) and 30% of dynamic eccentricity ($\delta_{de}$ = 0.3).

For building the analytical model of this motor, first the number *N* of intervals in which the air gap periphery is divided must be selected. In this work, it has been chosen N=3600, giving an angular resolution of $0.1^{\circ }$.

### 5.1. Analytical Model of the Tested IM in Healthy Conditions

#### 5.1.1. Primitive Inductance Tensor of the Healthy IM

**Figure 6:**
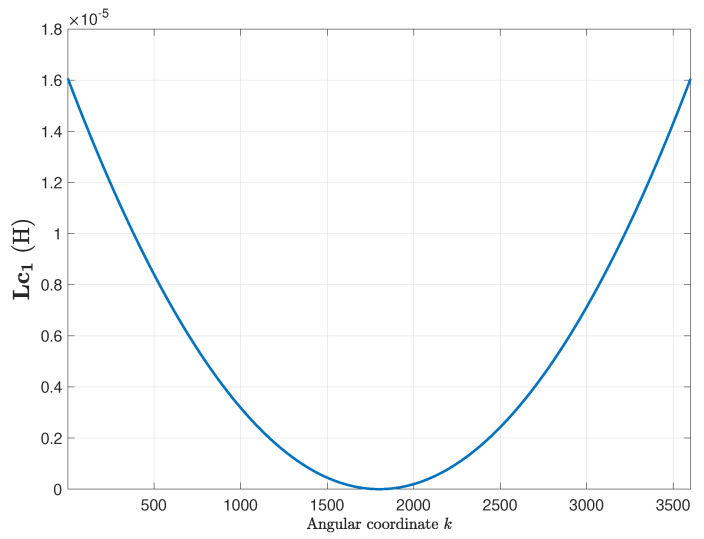
First column of the primitive inductance tensor (15), for the induction machine (IM) given in Appendix A. Each one of the rest of the columns is equal to the previous one, with its element rotated one position.

The primitive inductance tensor (15) has been computed for the healthy motor using (16), and its first column is represented in Figure 6. Each one of the rest of the columns of (15) is equal to the previous one, with its elements rotated by one position. This tensor is independent of the angular position of the rotor.

#### 5.1.2. Winding Tensor of the Healthy IM

**Figure 7:**
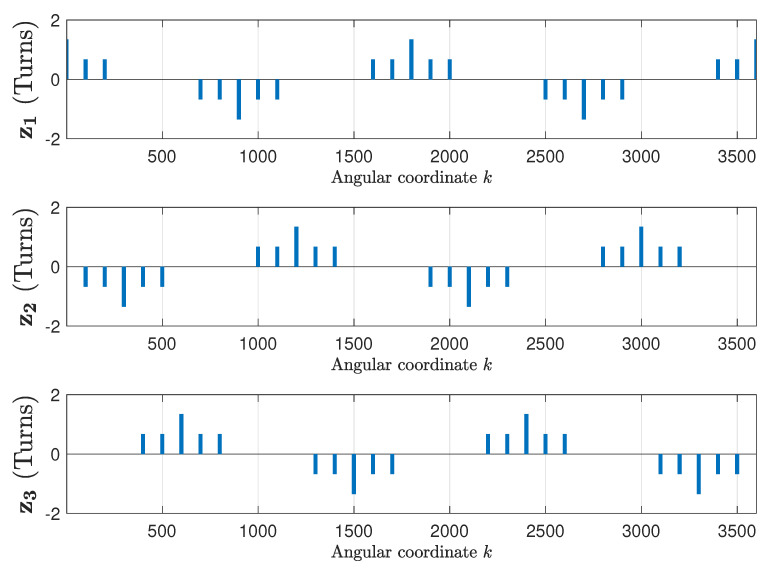
Columns 1–3 of the winding tensor (14), corresponding to the three stator phases of the IM given in Appendix A, which contains the current-sheet generated by each stator phase when fed by a unit current.

**Figure 8:**
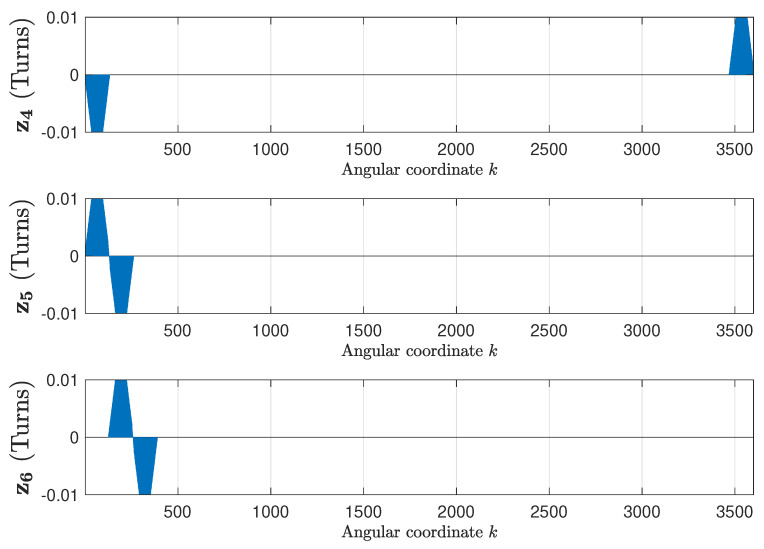
Columns 4–6 of the winding tensor (14), corresponding to the three first rotor loops of the IM given in Appendix A, which contains the current-sheet generated by each rotor loop when fed by a unit current.

The winding tensor, which contains the current-sheet generated by each phase, when fed with a unit current, has been obtained using the data provided in Appendix A. Figure 7 shows the three first columns of the winding tensor $C_{c}$ (14), corresponding to the stator phases, and Figure 8 shows the next three columns of $C_{c}$ (14), corresponding to the three first rotor loops. The inclination of rotors slots has been taken into account as in [49].

#### 5.1.3. Mutual Inductance Matrix of the Healthy IM

**Figure 9:**
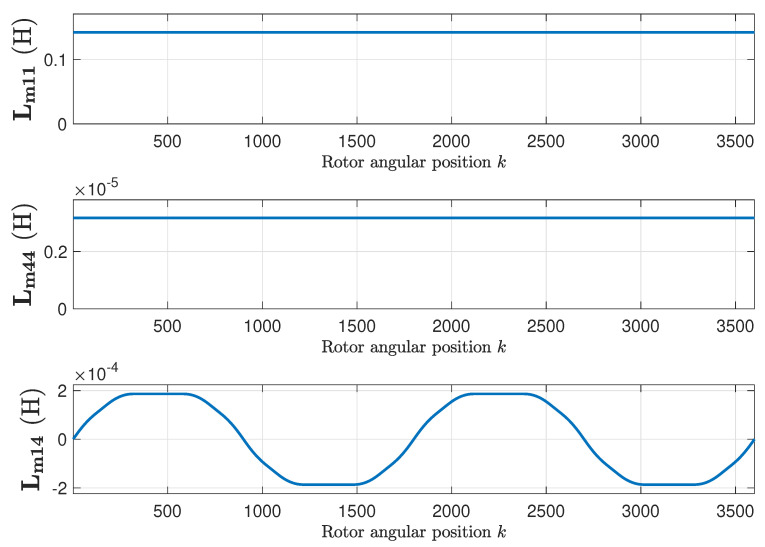
Self-inductance of the first stator phase (**top**), of the first rotor loop (**middle**), and mutual inductance between the first stator phase and the first rotor loop (**bottom**), as a function of the rotor position, for the healthy machine.

**Figure 10:**
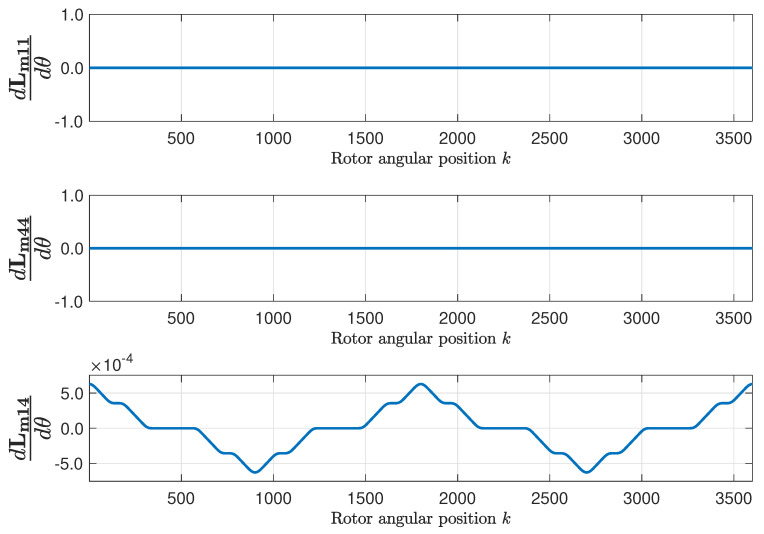
Angular derivatives of the self-inductance of the first stator phase (**top**), of the first rotor loop (**middle**), and mutual inductance between the first stator phase and the first rotor loop (**bottom**), as a function of the rotor position, for the healthy machine.

The mutual inductance matrix $L_{m}$ is found applying (15), using the primitive inductance tensor (Figure 9) and the winding tensor (Figure 7 and Figure 8). Figure 9 represents, as a function of the rotor position, the self inductance of the first stator phase (Figure 9, top), of the first rotor loop (Figure 9, middle), and the mutual inductance between the first stator phase and the first rotor loop (Figure 9, bottom). Figure 10 shows their respective angular derivatives.

#### 5.1.4. Resistance and Leakage Inductance Matrices of the Healthy Machine

The resistance (38) and leakage inductance (39) matrices of the healthy machine are assembled using (28), (29), (36) and (37), using the values of the resistance and the leakage inductance of a stator phase, a rotor bar and end ring segment given in Appendix A.

### 5.2. Analytical Model of the Tested IM with an Eccentricity Fault

The model of the motor given in Appendix A is obtained in this section assuming a mixed eccentricity fault, with a level of 30% of static eccentricity and 30% of dynamic eccentricity ($\delta_{se}$ = 0.3, $\delta_{de}$ = 0.3). It is modeled using the expression for the primitive inductance tensor of the eccentric IM (22).

#### 5.2.1. Primitive Inductance Tensor of the Eccentric IM

**Figure 11:**
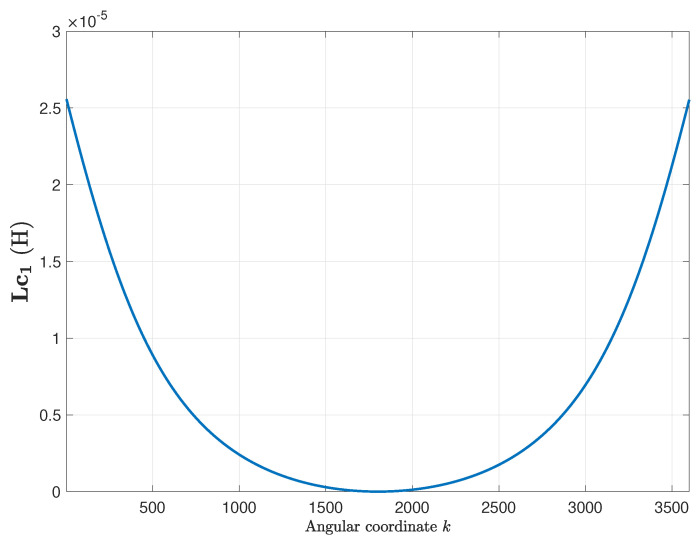
First column of the primitive inductance tensor (15), for the IM given in Appendix A with a mixed eccentricity fault. The other columns of this tensor cannot be obtained by a rotation of this column, due to the eccentricity of the rotor.

The primitive inductance tensor (15) of the eccentric IM has been computed using (22), using $\delta_{se}$ = 0.3 and $\delta_{de}$ = 0.3 in (18) and (19). Contrary to the case of the healthy machine, the primitive inductance tensor depends now on the rotor position. The columns of this tensor cannot be obtained by a rotation of the previous ones, due to the eccentricity of the rotor. Figure 11 shows the first column of the primitive inductance tensor, for a rotor angular position equal to zero. It is worth mentioning that the primitive inductance tensor just captures the eccentricity fault, being independent of the winding configuration.

#### 5.2.2. Winding Tensor of the Eccentric IM

The winding tensor of the healthy IM, shown in Figure 7 and Figure 8, is not affected by the eccentricity fault. Therefore, it is the same as the winding tensor of the healthy machine.

#### 5.2.3. Mutual Inductance Matrix of the Eccentric IM

**Figure 12:**
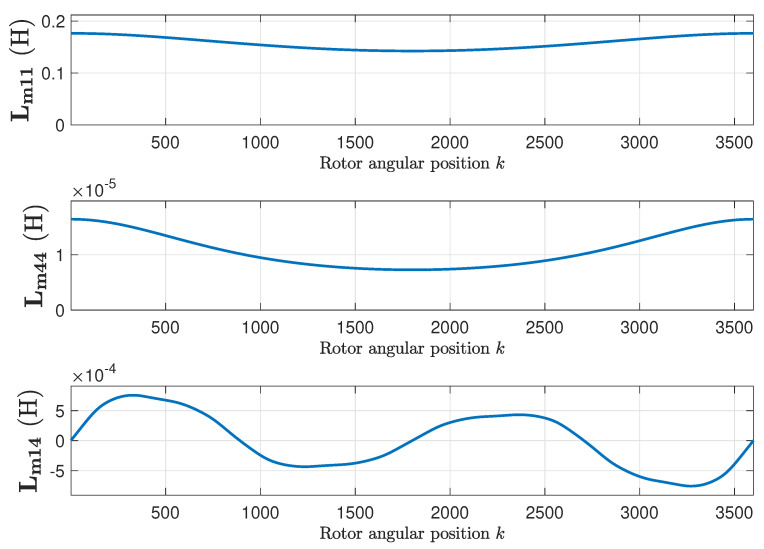
Self-inductance of the first stator phase (**top**), of the first rotor loop (**middle**), and mutual inductance between the first stator phase and the first rotor loop (**bottom**), as a function of the rotor position, for the eccentric machine.

**Figure 13:**
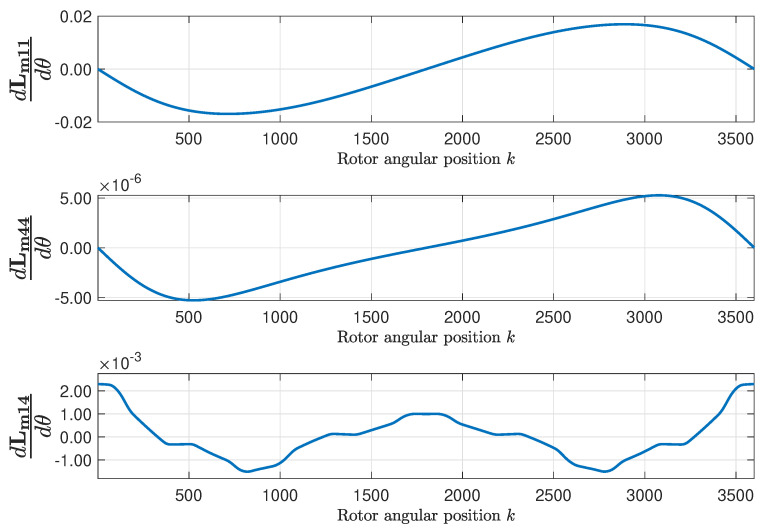
Angular derivatives of the self-inductance of the first stator phase (**top**), of the first rotor loop (**middle**), and mutual inductance between the first stator phase and the first rotor loop (**bottom**), as a function of the rotor position, for the eccentric machine.

The inductance matrix of the faulty IM is found by applying (15), using the primitive inductance tensor represented in Figure 12 and the winding tensor represented in Figure 7 and Figure 8. Figure 12 represents, as a function of the rotor position, the self inductance of the first stator phase (Figure 12, top), of the first rotor loop (Figure 12, middle), and the mutual inductance between the first stator phase and the first rotor loop (Figure 12, bottom). Figure 13 shows their respective angular derivatives.

#### 5.2.4. Resistance and Leakage Inductance Matrices of the Eccentric Machine

The resistance (38) and leakage inductance (39) matrices of the healthy machine are not affected by the eccentricity fault.

## 6. Experimental Validation

**Figure 14:**
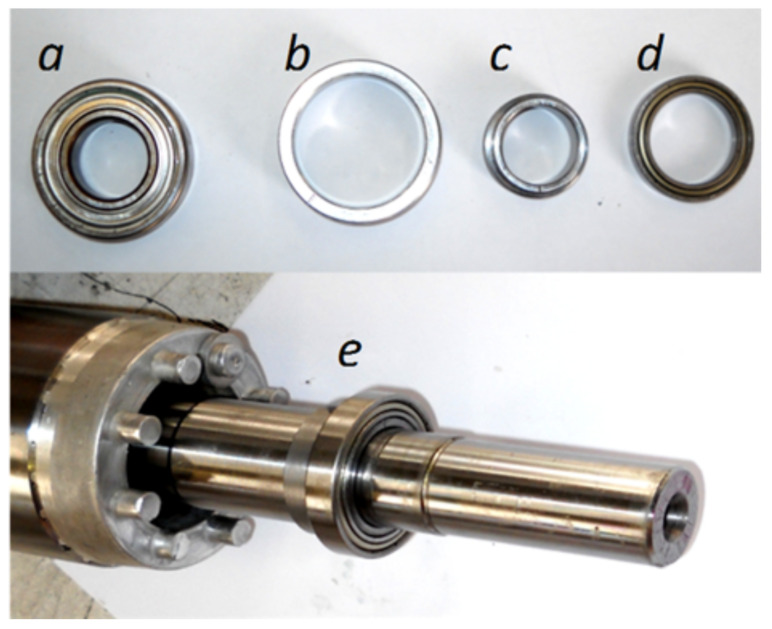
Rotor of the eccentric motor with a provoked mixed eccentricity fault. Top, from left to right: (**a**) original bearing, (**b**) external and (**c**) internal eccentric rings, and (**d**) new bearing. Bottom: (**e**) mounted unit on the shaft.

The experimental procedure carried out for the validation of the proposed method consists in provoking an artificial eccentricity fault to the tested motor (Appendix A), and comparing the characteristic fault harmonics that this fault induces in the motor current with those obtained from the simulated motor. To this end, the original bearings of the motor (see Figure 14a) have been replaced with new bearings (Figure 14d) having a smaller outer diameter and a greater inner diameter. These new bearings have been displaced from the center of the stator bore using two precision eccentric steel rings (Figure 14b,c), placed in the bearings housing (Figure 14b) and on the shaft (Figure 14c). The cylindrical surfaces of both rings are eccentric, 0.09 mm in the case of the outer ring b, and 0.09 mm in the case of the inner ring c. This assembly (Figure 14e) results in a rotor with a 30% of static eccentricity and a 30% of dynamic eccentricity.

A mixed eccentricity fault [51] induces two characteristic series of harmonic components in the motor current spectrum: one as side bands around the principal slot harmonics, and other one around the fundamental component. The frequencies of this low frequency series depend on the rotor speed, and can be obtained as

(40) $$f_{ME}\left(s\right)=f_{1}\pm \left(k\;\;\left(1-s\right)f_{1}/p\right)\;,\;\;\;\;\;k=1,2,3\ldots$$

where $f_{1}$ is the power supply frequency, *s* is the slip and *p* is the number of pole pairs of the machine.

Using the dominant component of the series (40) (*k* = 1), the mixed eccentricity fault can be detected through the presence in the stator current spectrum of harmonic components at frequencies:

(41) $$f_{ME}\left(s\right)=f_{1}\pm \left(1-s\right)f_{1}/p=f_{1}\pm f_{r},$$

where $f_{r}$ is the rotational frequency of the motor. For the tested motor (p=2), this gives

(42) $$f_{ME}\left(s\right)=f_{1}\pm \left(1-s\right)f_{1}/2$$

**Figure 15:**
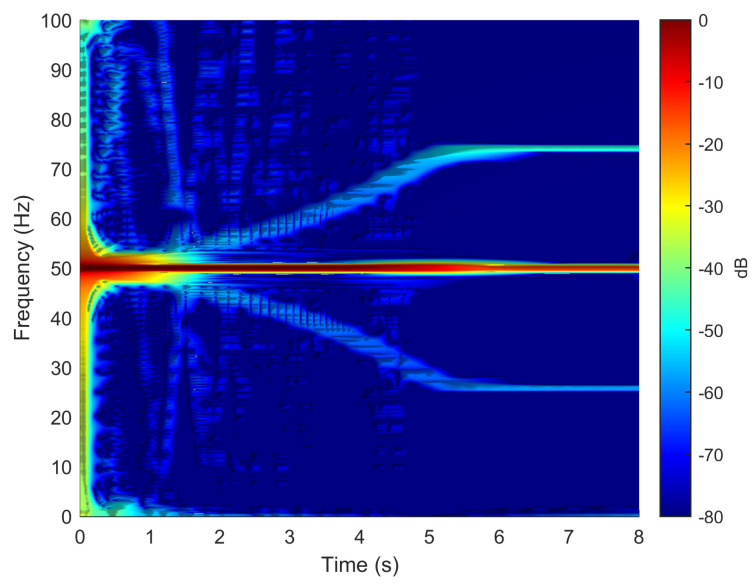
Spectrogram of the experimental current of the motor given in Appendix A, in faulty conditions, obtained during the start up transient. The eccentricity fault related components appear in this time-frequency plot, which indicates a faulty condition.

**Figure 16:**
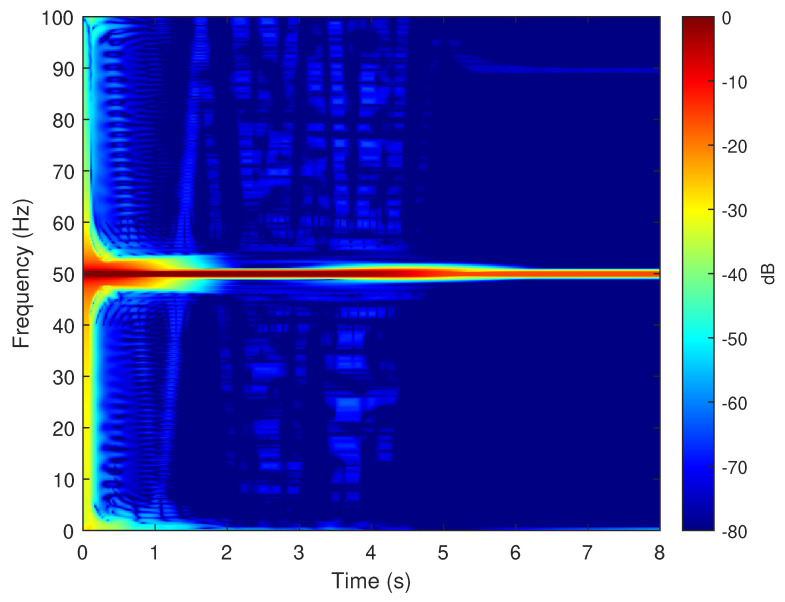
Spectrogram of the experimental current of the motor given in Appendix A, in healthy conditions, obtained during the start up transient. No eccentricity fault related components appear in this time-frequency plot, which indicates a healthy condition.

To verify the validity of the method proposed in this paper, in particular its ability to reproduce the fault harmonics at frequencies given by (42), the eccentric motor has been tested during a start-up transient, with a final permanent regime speed of 1445 rpm (s=(1500−1445)/1500=0.0367). To this end, one of the phase currents has been sampled, using the current clamp whose data is given in Appendix B, during an acquisition time of 8 seconds, with a sampling rate of 2 kHz. The spectrogram of this current, obtained with the computer platform given in Appendix C, is shown in Figure 15. As given by (42), two fault-related harmonics appear in permanent regime at frequencies $f_{ME}\left(0.0367\right)=50\pm \left(1-0.0367\right)\cdot 50/2=$ [25.92 Hz, 74.08 Hz]. Both harmonic components have the same frequency as the fundamental component at the beginning of the start-up transient, and their frequency varies proportionally to the speed up to their final frequency in the permanent regime. For comparison purposes, Figure 16 presents the spectrogram obtained during the start-up transient of the same motor in healthy conditions, prior to provoking the eccentricity fault. In this spectrogram there is no presence of the harmonic components produced by the mixed eccentricity fault.

**Figure 17:**
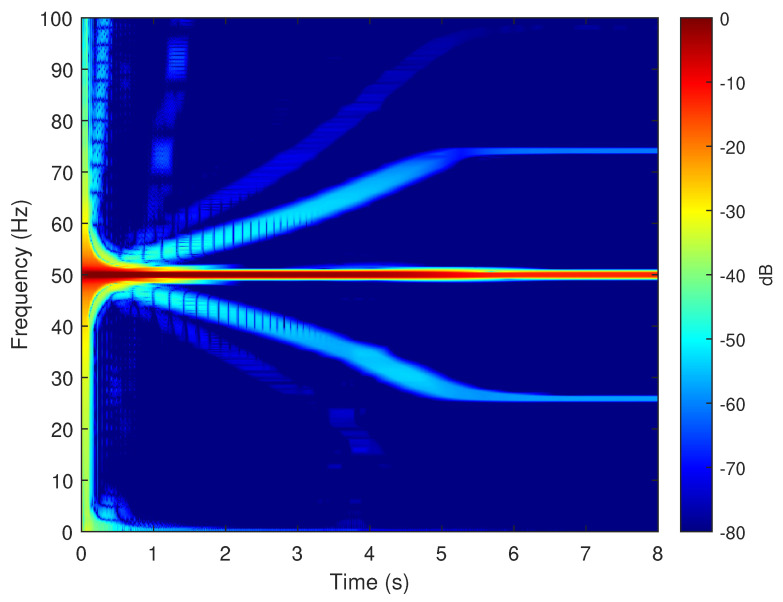
Spectrogram of the simulated current of the motor given in Appendix A, in faulty conditions, obtained during the start up transient. The eccentricity fault related components appear in this time–frequency plot, which indicates a faulty condition.

**Figure 18:**
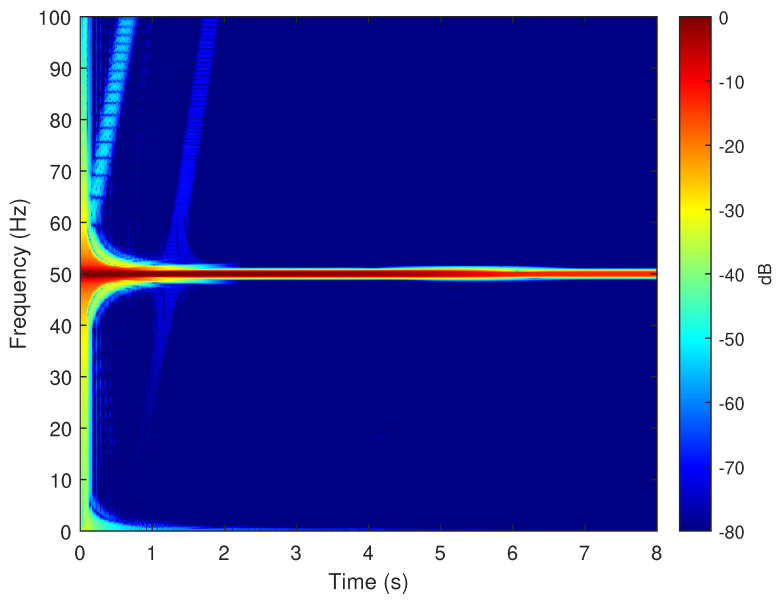
Spectrogram of the simulated current of the motor given in Appendix A, in healthy conditions, obtained during the start-up transient. No eccentricity fault-related components appear in this time-frequency plot, which indicates a healthy condition.

The motor given in Appendix A has been simulated under the same conditions as the experimental test, using the Simulink model given in Figure 1. The spectrogram of the simulated phase current, shown in Figure 17, displays correctly the characteristic signature of the eccentricity fault harmonics in the time-frequency plane, which assesses the validity of the method presented in this work. For comparison purposes, the motor has been simulated in healthy conditions, and in the spectrogram of its current, shown in Figure 18, there is no presence of the harmonic components produced by the mixed eccentricity fault.

## 7. Conclusions

Tensor algebra provides a powerful tool for developing the analytical model of IMs, because it allows us to gradually introduce the effects of any fault first at the conductor level, using the primitive inductance tensor, and after at the winding level, using the winding tensor. Routine procedures of tensor algebra facilitate the conversion of the primitive inductance tensor into the inductance matrix of the machine, for any winding configuration. In this work, this new method has been presented, applied to the development of the analytical model of an eccentric IM, and validated using experimental tests. The application of the winding tensor approach for building the analytical model of IMs with other types of faults is currently a work in progress.

## Appendix A. Commercial IM

Three-phase induction machine. Rated characteristics: P=1.1kW, f=50Hz, U=400/230V, I=2.7/4.6A, n=1410r/min, cosφ=0.8.

Machine dimensions: Effective length of the magnetic core = 120 mm, radius at the middle of the air gap = 54.1 mm, air gap length = 0.28 mm.

Stator: Three-phase winding, 36 slots, 78 wires/slot, winding pitch = 7/9, slot opening width = 2.1 mm, phase resistance 7.68 Ω, end winding leakage = 2.3 mH.

Rotor: Squirrel-cage winding, 28 bars, slot opening width = 1.4 mm, skew = one slot pitch, bar resistance = 0.00202 mΩ, end winding leakage = $2.45\times 10^{-5}$ mH.

## Appendix B. Current Clamp

Chauvin Arnoux MN60, Nominal measuring scope: 100 mA–20A, ratio input/output: 1 A/100 mV, intrinsic error: ≤ 2% + 50 mV, frequency use: 40 Hz–10 kHz.

## Appendix C. Computer Features

CPU: Intel Core i7-2600K CPU @ 3.40 GHZ RAM memory: 16 GB, Matlab Version: 9.7.0.1216025 (R2019b).
